# SARS-CoV-2-Specific Memory T Lymphocytes From COVID-19 Convalescent Donors: Identification, Biobanking, and Large-Scale Production for Adoptive Cell Therapy

**DOI:** 10.3389/fcell.2021.620730

**Published:** 2021-02-25

**Authors:** C. Ferreras, B. Pascual-Miguel, C. Mestre-Durán, A. Navarro-Zapata, L. Clares-Villa, C. Martín-Cortázar, R. De Paz, A. Marcos, J. L. Vicario, A. Balas, F. García-Sánchez, C. Eguizabal, C. Solano, M. Mora-Rillo, B. Soria, A. Pérez-Martínez

**Affiliations:** ^1^Hospital La Paz Institute for Health Research, IdiPAZ, University Hospital La Paz, Madrid, Spain; ^2^Hematology Department, University Hospital La Paz, Madrid, Spain; ^3^Histocompatibility, Centro de Transfusión de Madrid, Madrid, Spain; ^4^Research Unit, Basque Center for Blood Transfusion and Human Tissues, Osakidetza, Galdakao, Spain; ^5^Cell Therapy, Stem Cells and Tissues Group, Biocruces Bizkaia Health Research Institute, Barakaldo, Spain; ^6^Hospital Clínico Universitario de Valencia/Instituto de Investigación Sanitaria INCLIVA, Universidad de Valencia, Valencia, Spain; ^7^Infectious Diseases Unit, Internal Medicine Department, Hospital La Paz Institute for Health Research, IdiPAZ, University Hospital La Paz, Madrid, Spain; ^8^Instituto de Bioingeniería, Universidad Miguel Hernández de Elche, Alicante, Spain; ^9^Instituto de Investigación Sanitaria Hospital General y Universitario de Alicante (ISABIAL), Alicante, Spain; ^10^Pediatric Hemato-Oncology Department, University Hospital La Paz, Madrid, Spain; ^11^Faculty of Medicine, Universidad Autónoma de Madrid, Madrid, Spain

**Keywords:** memory T cells (Tmem), adoptive cell therapy (ACT), COVID-19, lymphopenia, biobank

## Abstract

Syndrome coronavirus 2 (SARS-CoV-2) pandemic is causing a second outbreak significantly delaying the hope for the virus’ complete eradication. In the absence of effective vaccines, we need effective treatments with low adverse effects that can treat hospitalized patients with COVID-19 disease. In this study, we determined the existence of SARS-CoV-2-specific T cells within CD45RA^–^ memory T cells in the blood of convalescent donors. Memory T cells can respond quickly to infection and provide long-term immune protection to reduce the severity of COVID-19 symptoms. Also, CD45RA^–^ memory T cells confer protection from other pathogens encountered by the donors throughout their life. It is of vital importance to resolve other secondary infections that usually develop in patients hospitalized with COVID-19. We found SARS-CoV-2-specific memory T cells in all of the CD45RA^–^ subsets (CD3^+^, CD4^+^, and CD8^+^) and in the central memory and effector memory subpopulations. The procedure for obtaining these cells is feasible, easy to implement for small-scale manufacture, quick and cost-effective, involves minimal manipulation, and has no GMP requirements. This biobank of specific SARS-CoV-2 memory T cells would be immediately available “off-the-shelf” to treat moderate/severe cases of COVID-19, thereby increasing the therapeutic options available for these patients.

## Introduction

The new severe acute respiratory syndrome coronavirus 2 (SARS-CoV-2) emerged as a worldwide pandemic in late 2019, causing an infectious disease known as COVID-19, with a wide and diverse range of symptoms. In most infected patients, the virus causes mild symptoms including fever and cough. In some cases, however, the virus causes a life-threatening disease with symptoms that include pneumonia, dyspnea and a hyperinflammatory process that includes cytokine storms and systemic immune thrombosis. Patients suffering from these symptoms require hospitalization and intensive treatment. A common feature of this severe disease is lymphopenia, which makes patients more vulnerable to co-infections and correlates with the severity disease (Qin et al., 2020; Zhao et al., 2020).

The first wave of the pandemic was contained with strong restrictive measures, social distancing, and healthcare interventions, although thousands of patients died. Far from disappearing, the SARS-CoV-2 pandemic has begun its second wave, thereby dimming the hopes for its complete eradication. The development of vaccines has vigorously pursued to generate active immunity through immunization (Thanh Le et al., 2020), but there is uncertainty as to the duration of the antibody-mediated immune response to COVID-19 (Li et al., 2008). Effective treatments are needed that can reduce symptoms severity and hospital stays and increase survival.

So far, the only treatment for COVID-19 is supportive. Antiviral therapy with lopinavir–ritonavir is ineffective in improving the outcomes hospitalized patients with COVID-19 (Cao and Hayden, 2020). Remdesivir has been recently approved to treat COVID-19, although its beneficial effect is still controversial (Jomah et al., 2020; Wang et al., 2020). Preliminary results with anti-inflammatory therapies such as dexamethasone (Recovery Collaborative Group et al., 2020) and mesenchymal stromal cells (Sanchez Guijo et al., 2020) have shown promising results for critically ill patients (World Health Organization [WHO] grade 6 and 7) (World Health Organization, 2020), but there are as of yet no effective antiviral therapies for stopping the progress of this disease in its early stages (WHO grade 1–4 moderate and severe) or even to prevent COVID-19.

The role of adaptive immunity in COVID-19 and the protective immunity conferred by T cells is still being characterized (Grifoni et al., 2020; Huang et al., 2020; Leung et al., 2020; Rodda et al., 2020; Sekine et al., 2020), and the role of memory T cells in conferring protection against SARS-CoV-2 has not yet been properly defined. The presence of memory T cells specific for another SARS coronavirus was found up to 11 years post-infection (Ng et al., 2016). This immunological memory creates a more rapid and robust secondary immune response to reinfections, which is determinant and constitutes the basis of adoptive cell therapy for viral infections in immunosuppressed patients in the context of allogeneic hematopoietic stem cell transplantation (HSCT). With this approach, the infusion of CD45RA^–^ memory T cells considerably reduces the morbidity and mortality induced by viral reactivations by, for example, cytomegalovirus (CMV) and Epstein Barr virus (EBV) and simultaneously reduces the alloreactivity conferred by naïve CD45RA^+^ T cells (Bleakley et al., 2014, 2015; Teschner et al., 2014; Triplett et al., 2015, 2018).

Memory T cells do appear when T cells recognize a pathogen presented by their local antigen-presenting cells. These T cells activate, proliferate, and differentiate into effector cells secreting compounds to control the infection. Once the pathogen has been cleared, most of the antigen-specific T cells disappear, and a pool of heterogeneous long-lived memory T cells persist (Mueller et al., 2013; Pennock et al., 2013). This population of memory T cells, defined as CD45RA^–^ or CD45RO^+^, is maintained over time conferring rapid and long-term immune protection against subsequent reinfections (Berard and Tough, 2002; Channappanavar et al., 2014).

In this study, we report the presence of a SARS-CoV-2 specific T-cell population within CD45RA^–^ memory T cells from the blood of convalescent donors that can be easily, effectively, and rapidly isolated by CD45RA depletion. These specific SARS-CoV-2 CD45RA^–^ memory T cells may be able to clear virally infected cells and confer T-cell immunity for subsequent reinfections. These cells can be stored for use in moderate and severe cases of COVID-19 patients requiring hospitalization, thereby representing an off-the-shelf living drug.

## Materials and Methods

### Donors’ Characteristics

The study included 6 COVID-19 convalescent donors and 2 healthy controls (Table 1). The convalescent donors were all tested for SARS-CoV-2 using reverse transcriptase polymerase chain reaction (RT-PCR) in nasopharyngeal samples between March and April 2020. The eligibility criteria included an age of 21–65 years, a history of COVID-19 with a documented positive RT-PCR test for SARS-CoV-2. At the time of this study, all donors tested negative for SARS-CoV-2. The median age of the convalescent donors was 37 years (range 23–41), 3 were women and 3 were men. The median duration until a negative PCR for SARS-CoV-2 was 13 days (range 5–17). Two of the donors presented with bilateral pneumonia but did not require hospitalization. Only 1 of the donors underwent treatment with oral hydroxychloroquine plus azithromycin plus lopinavir/ritonavir, while the other was treated with oral hydroxychloroquine plus azithromycin. The study enrolled two healthy donors who had not been exposed to COVID-19 patients and tested negative for anti-SARS-CoV-2 antibodies in June 2020. All participants granted their written consent, and the study was approved by the Hospital Institution Review Board (IRB number: 254/20).

**TABLE 1:** Participants’ characteristics.

Number of donors	6
Mean age, years (range)	37 (23–41)
Sex (female/male)	3/3
Time to SARS-CoV-2-negative PCR, days (range)	12.83 (5–17)
Bilateral pneumonia, *n*(%)	2 (33.3%)
Time until samples taken from negative PCR, days (range)	14.70 (7–33)
Outpatients, *n* (%)	6 (100%)
**Treatment**	
Acetaminophen	4
Hydroxychloroquine + azithromycin + lopinavir/ritonavir	1
Hydroxychloroquine + azithromycin	1

### Cell Processing and Detection of SARS-CoV-2-Specific Memory T Cells by Interferon-Gamma Assay

Peripheral blood mononuclear cells (PBMCs) from healthy donors and convalescent donors were isolated from their peripheral blood by density gradient centrifugation using Ficoll-Paque (GE Healthcare, Chicago, IL, United States). Briefly, the cells were rested overnight (o/n) at 37°C in TexMACS Medium (Miltenyi Biotec, Bergisch Gladbach, Germany) supplemented with 10% AB serum (Sigma-Aldrich, Saint Louis, MO, United States) and 1% penicillin/streptomycin (Sigma-Aldrich). The following day 1 × 10^6^ cells were stimulated with pooled or individual overlapping SARS-CoV-2 peptides at a final concentration of 0.6 nmol/mL. For the positive control, 1 × 10^6^ cells were stimulated in the presence of the plate-bound stimulator OKT3 at a final concentration of 2.8 μg/mL (mouse anti-human CD3 Clone OKT3, BD Biosciences). Cells with SARS-CoV-2 peptides and positive control were co-stimulated with CD28/CD49d at a final concentration of 5 μg/mL (anti-human CD28/CD49d Purified Clone L293 L25, BD Biosciences). Basal interferon gamma (IFN-γ) production by PBMCs was included as a background control in the absence of stimulation and co-stimulation. The peptide pools were short 15-mer peptides with 11 amino acid overlaps that can bind MHC class I and class II complexes and were therefore able to stimulate CD4^+^ and CD8^+^ T cells. The peptides cover the immunodominant sequence domains of the surface glycoprotein S, the complete sequence of the nucleocapsid phosphoprotein N and the membrane glycoprotein M (GenBank MN908947.3, Protein QHD43416.1, Protein QHD43423.2, Protein QHD43419.1; Miltenyi Biotec, Germany). After 5 h of stimulation, the cells were labeled with IFN-γ Catch Reagent (IFN-γ Secretion Assay-Detection Kit, human Miltenyi Biotec) containing bispecific antibodies for CD45 and IFN-γ, which were secreted by the stimulated target cells. After the secretion phase, the cell surface-bound IFN-γ was targeted using the IFN-γ PE antibody included in the kit.

### Phenotype of Memory T Cells Containing SARS-CoV-2-Specific T Cells Determined by Flow Cytometry Assay

We analyzed the cell composition of the T cells specifically activated by SARS-CoV-2 in the IFN-γ assay by subtracting the basal cytokine response from the background control. We stained the cell surface for 20 min at 4°C using the following fluorochrome-conjugated antibodies titrated to their optimal concentrations: CD45RA FITC (BD Pharmingen), CD27 APC (BD Pharmingen), CD3 VioGreen (Miltenyi Biotec), CD4 PECy7 (BD Pharmingen), CD8 APC Cy7 (BD Pharmingen), and 7AAD (BD Horizon). For the Treg panel CD25 BV421 (BD Horizon) and CD127 PE-CF594 (BD Horizon), were used. For the activation panel, HLA-DR BV 421 (BD Pharmingen), CD69 BV421 (Biolegend), and CD25 BV421 (BD Horizon), were used. For the exhaustion panel PD1 AF700 (Biolegend) and NKG2A BV421 (Biolegend) were used. For the chemokine panel, CD103 BV421 (BD Horizon) and CCR7 PE-CF594 (BD Horizon) were used. Cell acquisition was performed using a Navios cytometer (Beckman Coulter), acquiring an average of 200,000 cells. The analysis was performed using FlowJo 10.7.1 (FlowJo LLC).

### Interleukin-15 Stimulation of Memory T Cells

CD45RA^–^ memory T lymphocytes from the convalescent donor were thawed and stimulated with interleukin (IL)-15 to obtain an activated phenotype. Cells were incubated in TexMACS Medium (Miltenyi Biotec, Germany) supplemented with 5% AB serum (Sigma-Aldrich, Saint Louis, MO, United States), 1% penicillin/streptomycin (Sigma-Aldrich, Saint Louis, MO, United States) and 50 ng/mL of IL-15 o/n and for 72 h. After that time the cells were harvested and the phenotypic assay was performed. The same culture without IL-15 was run in parallel as a control.

### Donor Selection, Human Leukocyte Antigen Typing, and Large Clinical Scale CD45RA^+^ T Cell Depletion

The criteria for selecting convalescent donors were as follows: (1) IFN-γ secretion upon activation with the three SARS-CoV-2-specific peptides (M, N, S) and, (2) the most frequent human leukocyte antigen (HLA) typing to cover most of the population. The HLA phenotype of the convalescent donor was performed at the Centro de Transfusión of the Comunidad of Madrid on two independent samples by SSO and NGS: A^∗^02:01,A^∗^24:02/B^∗^44:02,B^∗^51:01/C^∗^16:02,C^∗^16:04/DRB1^∗^07:01,DRB1^∗^11:03/DQB1^∗^02:02,DQB1^∗^03:01.

Non-mobilized apheresis was performed at the Bone Marrow Transplantation and Cell Therapy Unit of University Hospital La Paz (Madrid, Spain) using a CliniMACS Plus cell separation system (Miltenyi Biotec). The donor provided written informed consent, and the study was conducted according to the Declaration of Helsinki protocol and the guidelines of the local ethics committee (IRB number 5579). The Unit was responsible for complying with the requirements regarding the quality and safety of the donation, obtention, storage, distribution, and preservation of human cells and tissues under the Spanish specific regulation. Following apheresis, CD45RA^+^ cells were depleted by immunomagnetic separation using a CliniMACS CD45RA Reagent and the CliniMACS Plus system (both from Miltenyi Biotec), following the manufacturer’s instructions. CD45RA^–^ cells were frozen using autologous plasma plus 5% dimethyl sulfoxide (DMSO) and stored. We were able to cryopreserve 30 aliquots at various doses according to the trial design. The viability, purity, phenotype and spectratyping of the CD45RA^–^ fraction were analyzed by flow cytometry (FCM).

### TCR Spectratyping

Most of the CDR3-encoding regions of the TCRV- and TCRV-γ genes were amplified using 2 V-J multi-primer sets for each locus and one additional multi-primer set covering D-J TCRV-β (Vitro, Master Diagnostica, Spain). Primers marked at their 5′ end with 6-FAM fluorochrome enabled the denatured fragment size analysis by capillary electrophoresis (ABI3130 DNA-analyzer) and Genemapper software (Thermo Fisher Scientific, United States).

### Statistical Analysis

The quantitative variables are expressed as mean ± standard deviation (SD), while the qualitative variables are expressed as percentages (%). A two-tailed Mann–Whitney non-parametric test was used for comparison means for the non-paired samples using GraphPad Prism (version 8.0.0 for Windows, GraphPad Software, San Diego, CA, United States). A *P*-value < 0.05 was considered statistically significant.

## Results

### Memory T Cells From Convalescent Donors Contain a SARS-CoV-2-Specific Population

**FIGURE 1:**
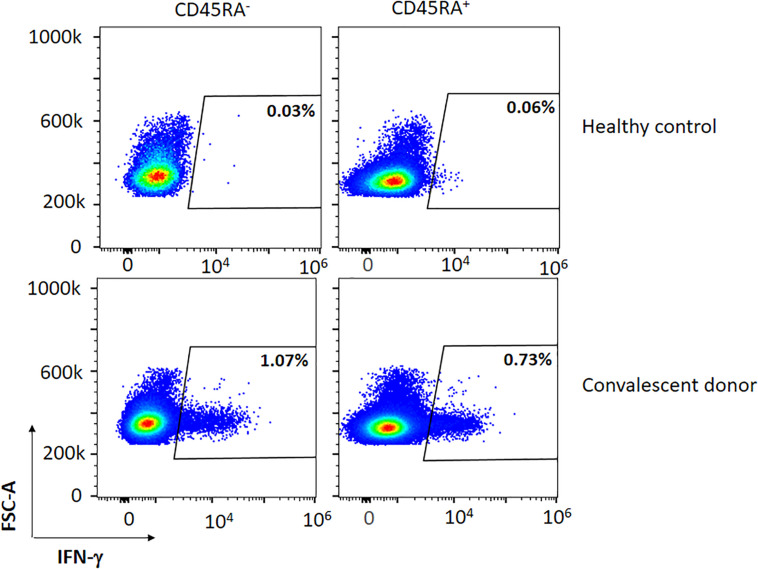
Representative figure of the expression of IFN-γ in a convalescent and healthy donor within the CD45RA^+^ and CD45RA^–^ subpopulations after co-culture with the mixture of the three peptides (M, N, S).

We detected the presence of a SARS-CoV-2-specific population in both subsets of naïve CD45RA^+^ and memory CD45RA^–^ T cells in the PBMCs of the convalescent donors but not in the healthy controls (Table 2 and Figure 1). The subsets also showed reactivity for the single peptides M, N, and S (data not shown). The mean CD45RA^–^CD3^+^ population in the convalescent donors was 90.01%. IFN-γ expression within the CD45RA^–^CD3^+^ population was 1.12%, whereas IFN-γ expression within the CD45RA^+^CD3^+^ population was 0.40% (*P* = 0.065) (Table 2). We detected no IFN-γ expression in the healthy individuals. Despite the cohort’s small size, we found no synergistic effect on the percentage of IFN-γ when the three peptides were mixed when compared with the single peptides (data not shown).

**TABLE 2:** Immunophenotypic characterization of the healthy controls and the convalescent donors after co-culture with the mixture of the three SARS-CoV-2 peptides (M, N, S).

Cell type	Subpopulation	C1	C2	Mean Pepx3	SD	D1	D2	D3	D4	D5	D6	Mean Pepx3	SD
CD45RA^–^	% CD45RA^–^	37.68	22.17	29.93	10.97	31.20	24.07	18.16	43.90	19.90	33.24	28.41	9.67
	IFN-γ+	0.00	0.00	0.00	0.00	0.77	0.99	3.00	0.30	0.90	0.87	1.14	0.94
CD45RA^–^ CD3+	% CD3+	61.69	78.38	70.04	11.80	93.84	93.45	94.53	77.12	86.64	94.48	90.01	6.99
	IFN-γ+	0.00	0.00	0.00	0.00	0.69	0.79	3.03	0.34	1.01	0.83	1.12	0.96
CD45RA^–^ CD8+	% CD8+	17.14	9.97	13.56	5.07	17.02	22.17	16.27	26.62	11.88	35.07	21.51	8.37
	IFN-γ+	0.00	0.00	0.00	0.00	0.38	0.46	3.45	0.12	0.70	0.00	0.85	1.29
	% CD8 + CM	87.86	87.29	87.58	0.40	86.52	65.72	76.30	88.00	90.01	42.50	74.84	18.28
	IFN-γ+	0.00	0.00	0.00	0.00	0.31	0.33	2.88	0.11	0.64	0.48	0.79	1.04
	% CD8 + EM	12.06	12.67	12.37	0.43	13.44	34.25	23.70	11.94	9.85	57.47	25.11	18.30
	IFN-γ+	0.00	0.00	0.00	0.00	0.00	0.79	5.58	0.00	0.00	0.00	1.06	2.23
CD45RA^–^ CD4+	% CD4+	68.15	86.69	77.42	13.11	75.99	75.83	79.31	70.60	80.07	60.63	73.74	7.24
	IFN-γ+	0.00	0.00	0.00	0.00	0.81	0.91	2.93	0.43	1.11	1.32	1.25	0.87
	% CD4 + CM	89.09	81.50	85.30	5.37	70.76	86.05	90.34	82.88	91.94	90.78	85.46	7.96
	IFN-γ+	0.00	0.00	0.00	0.00	0.56	1.01	2.93	0.47	1.13	1.44	1.26	0.89
	% CD4 + EM	10.87	18.47	14.67	5.37	29.23	13.94	9.66	17.10	8.01	9.22	14.53	7.97
	IFN-γ+	0.00	0.00	0.00	0.00	1.37	0.39	3.17	0.21	1.35	0.98	1.25	1.06
CD45RA+	% CD45RA+	61.91	77.83	69.87	11.26	68.69	75.86	81.86	55.98	80.30	66.75	71.57	9.74
	IFN-γ+	0.00	0.02	0.01	0.00	0.67	0.93	6.47	0.10	0.52	0.32	1.50	2.45
CD45RA + CD3+	% CD3+	70.44	78.05	74.25	5.38	52.93	79.05	60.97	59.8	74.60	79.16	67.75	11.26
	IFN-γ+	0.02	0.01	0.02	0.00	0.40	0.27	1.25	0.06	0.23	0.17	0.40	0.43
CD45RA + CD8+	% CD8+	22.77	24.35	23.56	1.12	56.52	27.24	49.85	32.1	33.08	38.60	39.57	11.34
	IFN-γ+	0.00	0.00	0.00	0.00	0.13	0.46	1.69	0.09	0.41	0.21	0.50	0.60
	% CD8 + Naive	72.84	83.57	78.21	7.59	76.57	84.64	80.40	95.9	80.80	44.83	77.19	17.19
	IFN-γ+	0.00	0.00	0.00	0.00	0.17	0.22	0.51	0.10	0.29	0.15	0.24	0.15
	% CD8 + TEMRA	27.14	16.43	21.79	7.57	23.43	15.35	19.60	4.1	19.20	55.17	22.81	17.19
	IFN-γ+	0.00	0.00	0.00	0.00	0.07	1.81	7.02	0.00	1.37	0.29	1.76	2.68
CD45RA + CD4+	% CD4+	70.26	70.09	70.18	0.12	32.35	70.30	47.27	65.88	59.50	54.40	54.95	13.75
	IFN-γ+	0.00	0.00	0.00	0.00	0.47	0.14	0.55	0.06	0.22	0.09	0.26	0.21
	% CD4 + Naive	99.53	98.72	99.13	0.57	95.67	99.53	99.32	99.57	99.35	99.21	98.78	1.53
	IFN-γ+	0.00	0.00	0.00	0.00	0.21	0.13	0.52	0.07	0.21	0.09	0.21	0.16
	% CD4 + TEMRA	0.47	1.28	0.88	0.57	4.33	0.47	0.68	0.43	0.65	0.79	1.23	1.53
	IFN-γ+	0.00	0.00	0.00	0.00	5.77	0.00	2.30	0.00	0.00	0.00	1.35	2.35

*The mean value after 5 h of exposure for the three SARS-CoV-2 peptides (M,N,S) is shown. SD, standard deviation; C, healthy controls; D, convalescent donors; Pepx3, mixture of the single SARS-Cov-2 peptides M,N,S.*

### Identification of SARS-CoV-2-Specific CD4^+^ T and CD8^+^ T-Cell Responses Within CD45RA^–^ Memory T Cells

**FIGURE 2:**
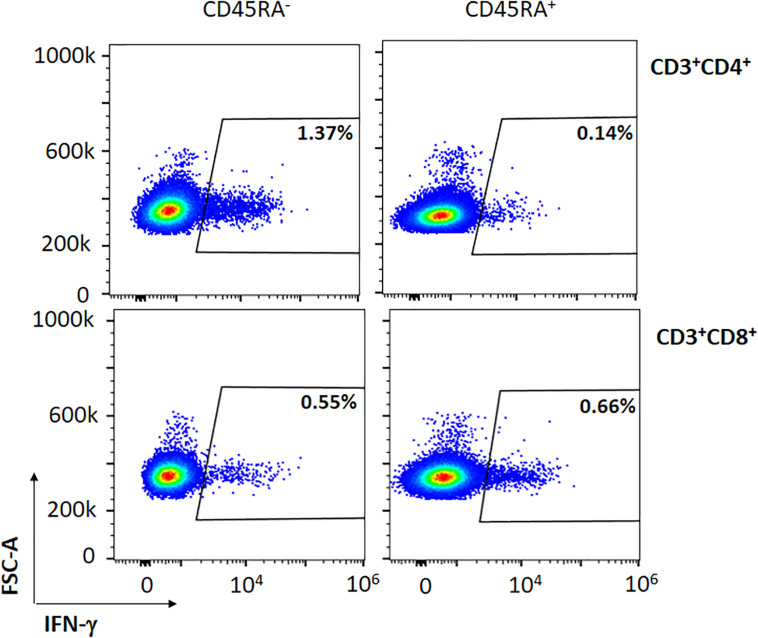
Representative figure of the expression of IFN-γ in a convalescent donor within the CD45RA^–^ CD3^+^CD4^+^ and CD45RA^–^ CD3^+^CD8^+^ subpopulations after co-culture with the mixture of the three peptides (M, N, S).

We then sought to determine whether both CD8^+^ and CD4^+^ subsets contained specific SARS-CoV-2 T cells within the PBMCs of the convalescent donors. Among all subsets studied, we observed that CD45RA^–^CD4^+^ and CD45RA^–^CD8^+^ cells expressed 1.25 and 0.85% of IFN-γ, respectively (*P* = 0.132) (Table 2, Figure 2, and data not shown). Thus, all of the convalescent donors who recovered from COVID-19 generated CD4^+^ T and CD8^+^ T-cell responses against SARS-CoV-2 within the memory CD45RA^–^ T-cell population.

**FIGURE 3:**
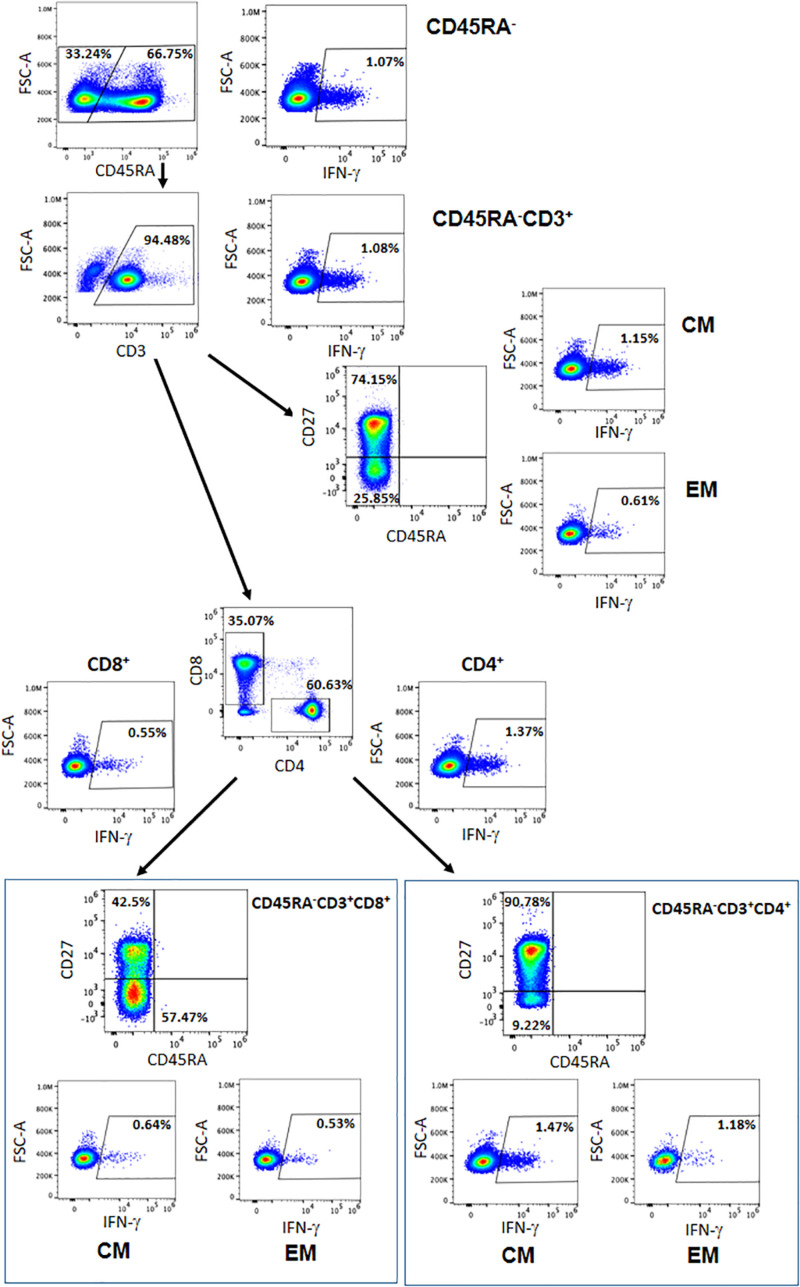
Representative figure of the gating strategy of the different cells subsets within the CD45RA^–^ population and the IFN-γ expressed. CM = central memory, EM = effector memory.

We then analyzed the T central memory (CM) (CD45RA^–^CD3^+^CD27^+^) and T effector memory (EM) (CD45RA^–^CD3^+^CD27^–^) compartments. Although there were no significant differences, we observed responses to the SARS-CoV-2-specific peptides within all subpopulations. Within the CD4^+^ and CD8^+^ CM T-cell subsets, we detected a mean of 1.26 and 0.79% of IFN-γ^+^ cells, respectively (*P* = 0.132). When examining the CD4^+^ and CD8^+^ EM T-cell subsets, we detected a mean of 1.25 and 1.06% of IFN-γ^+^ cells, respectively (*P* = 0.108) (Table 2 and Figure 3). These data demonstrate the presence of a population of memory T cells specific for SARS-CoV-2 within the CD45RA^–^CD3^+^ memory T cells.

### Large-Scale CD45RA Depletion. Creation of an Off-the-Shelf Biobank of CD45RA^–^ Memory T Cells From a COVID-19 Convalescent Donor

After depletion of the CD45RA^+^ cells (as described in the Materials and Methods section), 99.8% of the cells were CD45RA^–^CD3^+^, and most of the CD45RO^+^ cells were CD4^+^ with a high CD4/CD8 ratio. The viability of the cells after thawing was 98–99% (data not shown).

### Identification of SARS-CoV-2-Specific CD4^+^ T and CD8^+^ T-Cell Responses Within CD45RA^–^ Memory T Cells After CD45RA Depletion

After CD45RA depletion, the percentage of CD45RA^–^CD3^+^ cells were 80.05%. Within that population, 83.56% of the cells were CD4^+^ and 14.37% were CD8^+^ T cells. After exposure to the three SARS-CoV-2-specific peptides, the CD3^+^, CD3^+^CD4^+^, and CD3^+^CD8^+^ subsets expressed IFN-γ at a rate of 0.36, 0.38, and 0.31%, respectively (Table 3).

**TABLE 3:** Phenotype of CD45RA memory T cells from a convalescent donor after large scale CD45RA depletion.

	Cell marker	% of cells after thawing	% IFN-γ^+^	Fold change IL-15 (o/n)*	Fold changeIL-15 (72 h)**
CD45RA^–^ subpopulation	CD45RA^–^	99.85	0.36	1.00	0.99
	CD45RA^–^CD3^+^	80.05	0.36	1.05	0.99
	CD45RA^–^CD3^+^CD4^+^	83.56	0.38	1.02	0.98
	CD45RA^–^CD3^+^CD8^+^	14.37	0.31	0.84	1.10
	CD45RA^–^CD4^+^				
	CD27^+^ (CM)	89.13	0.38	1.01	1.02
	CD27^–^ (EM)	10.86	0.39	0.94	0.86
	CD127lowCD25^+^ (Treg)	11.26	N/A	1.27	2.03
	CD45RA^–^ CD8^+^				
	CD27^+^ (CM)	61.65	0.70	1.07	1.03
	CD27^–^ (EM)	38.34	0.00	0.89	0.95
T-cell activation marker	CD3^+^				
	HLA^–^DR^+^	19.40	0.93	1.15	2.63
	CD69 BV421 high	0.46	0.00	3.18	29.59
	CD25^+^	60.68	N/A	1.50	8.52
	CD4^+^				
	HLA-DR^+^	16.70	0.70	1.16	2.42
	CD69 BV421 high	0.38	0.00	2.90	59.75
	CD25^+^	66.10	N/A	1.48	8.58
	CD8^+^				
	HLA-DR^+^	29.19	1.21	1.15	1.89
	CD69 BV421 high	0.31	1.04	2.77	5.64
	CD25^+^	9.60	N/A	4.42	36.42
T-cell exhaustion markers	CD3^+^				
	NKG2A^+^	1.88	0.00	0.73	1.39
	PD1 AF700	0.70	0.75	0.71	8.70
	CD4^+^				
	NKG2A^+^	0.14	0.00	1.24	0.56
	PD1 AF700	1.43	0.62	0.84	9.15
	CD8^+^				
	NKG2A^+^	10.24	0.00	0.76	1.22
	PD1 AF700	0.13	0.00	1.69	6.14
Chemokine receptor and integrin	CD3^+^				
	CCR7	81.00	N/A	1.03	1.00
	CD103	2.18	N/A	0.74	1.24
	CD4^+^				
	CCR7	88.13	N/A	1.00	0.97
	CD103	1.38	N/A	0.74	0.99
	CD8^+^				
	CCR7	45.42	N/A	1.48	1.40
	CD103	5.96	N/A	0.89	1.39

*Fold change increase after overnight (o/n) treatment with IL-15. **Fold change increase after 72 h with IL-15. Fold change regarding the receptor expression has been calculated as the ratio between the value with IL-15 and the value from a control without IL-15. N/A,not available.

Most of the CD4^+^ and CD8^+^ cells had a CM phenotype (89.1 and 61.6%, respectively). Both the CM and EM subpopulations expressed IFN-γ after exposure to the three peptides. Thus, we found that 0.38, 0.70, and 0.39% of the cells within the CD4^+^ CM, CD8^+^ CM, and CD4^+^ EM subsets expressed IFN-γ, respectively. We found no specific IFN-γ expression within the CD8^+^ EM subset (Table 3).

We then characterized in-depth the phenotypic expression of the activation, exhaustion, and Treg markers within the CD45RA^–^ population of the convalescent donor. The percentage of Treg defined by CD127^*low*^CD25^+^ was 11.26%. We found that the CD45RA^–^CD3^+^ cells expressed the activation marker HLA-DR (19.40%) in both subsets of CD4^+^ (16.70%) and CD8^+^ cells (29.19%). After exposure to the three SARS-CoV-2-specific peptides, we observed that 0.93, 0.70, and 1.21% of the cells within the CD3^+^HLA^–^DR^+^, CD4^+^HLA^–^DR^+^, and CD8^+^HLA-DR^+^ populations expressed IFN-γ, respectively. The CD45RA^–^CD3^+^ cells also expressed the exhaustion marker NKG2A (1.88%), and this expression was 10.24% within the CD8^+^ subset, whereas it was nearly undetectable in the CD4^+^ cells (0.14%). We found no IFN-γ expression within the NKG2A^+^ population. In addition, 0.70% of the CD45RA^–^CD3^+^ cells expressed the exhaustion marker PD-1, and this expression was 1.43% within the CD4^+^ subset and 0.13% within the CD8^+^ cells. We found 0.62% of IFN-γ^+^ cells within the CD45RA^–^CD3^+^CD4^+^PD-1^+^ population after exposure to the three peptides (Table 3).

### CDR3 Use of TCR

**FIGURE 4:**
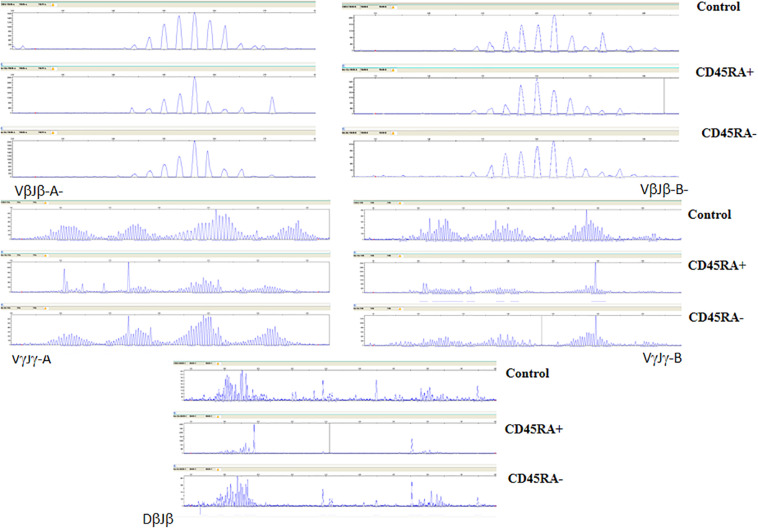
TCR-β and -γ spectratyping for five multi-primer sets covering most of the CDR3-encoding regions. Electropherograms showing Gaussian distribution of polyclonal population (Control) is also mainly seen in CD45RA^–^, with oligoclonal patterns in CD45RA^+^. Some predominant fragments are shared in both convalescent donor populations.

The polyclonal distribution for both TCR-β and TCR-γ CDR3-encoding regions was almost identical among the controls and the CD45RA^–^ population, whereas different oligoclonal fragments were observed in the CD45RA^+^ population. Three of the oligoclonal fragments seen in the CD45RA^+^ cells were also identified in the CD45RA^–^ cells (Figure 4).

### Induction of an Activated Memory T-Cell Phenotype Within CD45RA^–^ Memory T Cells After CD45RA Depletion

IL-15 is an essential cytokine for memory T cells that induces the activation, proliferation, and survival of T cells. After incubating the CD45RA^–^ T cells with IL-15, we observed an increase in the activation markers HLA-DR, CD69, and CD25 after 72 h of incubation (2.63-fold, 29.59-fold, and 8.52-fold, respectively) when compared with the o/n incubation (1.15-fold, 3.18-fold, and 1.50-fold) (Table 3). The expression of the exhaustion markers NKG2A and PD-1 was also higher after 72 h of incubation (Supplementary Figures 1–5). We then examined the expression of chemokine CCR7 and integrin CD103, which are important for the homing of T-cells to the respiratory tract (Campbell et al., 2001; Uss et al., 2006). We observed that most of the CD3^+^CD4^+^ cells expressed CCR7^+^ cells (88.13%), whereas the CD3^+^CD8^+^ subpopulation expressed 45.42% of CCR7^+^ cells. As expected, CD103 expression was low in the peripheral blood (Uss et al., 2006). We detected an expression of 2.18, 5.96, and 1.38% in the CD3^+^CD103^+^, CD3^+^CD8^+^CD103^+^, and CD3^+^CD4^+^CD103^+^ compartments, respectively. Although the fold increase was not particularly remarkable in the CD45RA^–^CD3^+^ cells after 72 h of incubation (1.00-fold CCR7 and 1.24-fold CD103), the increase was higher (1.40) within the CD8^+^ subpopulation (Table 3 and Supplementary Figures 6,7).

## Discussion

In the absence of an effective vaccine and with the emergence of a second wave, there is an urgent need to find effective treatments for COVID-19. Here we report the presence of a SARS-CoV-2-specific T-cell population within the CD45RA^–^ memory T cells of blood from convalescent donors. These cells can be easily, effectively, and rapidly isolated following a donor selection strategy based on IFN-γ expression after exposure with SARS-CoV-2-specific peptides and HLA antigen expression, thereby obtaining clinical-grade CD45RA^–^ memory T cells from the blood of convalescent donors. These cells can be biobanked, thawed, and employed as a treatment for moderate to severe cases of COVID-19. These so-called “living drugs” retain the memory against SARS-CoV-2 and other pathogens the donors have encountered. Unlike plasma, where the concentration decreases after infusion, memory T cells expand and proliferate and should therefore have a more lasting effect.

In previous studies, this population of CD45RA^–^ memory T cells showed no alloreactivity when compared with the CD45RA^+^ counterpart (Fernández et al., 2017). These cells were mainly CD4+ (Fernández et al., 2019) and showed effectiveness against viral infections (Triplett et al., 2015). Phenotypically, we found that CD45RA^–^ memory T cells were fully capable of producing IFN-γ in the presence of SARS-CoV-2-specific peptides. Both CD4^+^ and CD8^+^ CM and EM subsets were able to generate IFN-γ after exposure to the SARS-CoV-2 peptides, showing coverage of response. CD8^+^ cytotoxic cells can kill virally infected cells by secreting cytokines; at the same time, CD4^+^ T cells increase the ability of CD8^+^ T cells to eliminate the virus. They have been shown to play an important role in controlling the viral replication of other viruses such as EBV and CMV (Juno et al., 2017). EM T cells are the first responders to infection, with a quick and strong response to pathogens, whereas CM T cells proliferate and create a new round of effector T cells (Pennock et al., 2013; Farber et al., 2014). IL-15 is essential for the survival of memory CD8^+^ and CD4^+^ T-cell subsets, promoting the activation of CD4^+^T cells, cytokine production and proliferation, and the maintenance of the memory population (Brincks and Woodland, 2010; Chen et al., 2014). After incubating the cells for 3 days with IL-15, we obtained a phenotype characteristic of an activated state, as shown by the fold increase in the activation markers HLA-DR, CD69, and CD25 and in the CCR7 and CD103 markers characteristic of the homing of T cells to the lymph nodes and mucosal tissues.

In our study, we observed no IFN-γ production by the SARS-CoV-2-specific T cells in healthy unexposed individuals, which agrees with the findings of Peng et al. (2020) but differs from other previously published data (Grifoni et al., 2020). This discrepancy could be due to the different detection methods employed and the small sample size.

Studies have shown the correlation between neutralizing antibodies and symptom severity, where antibody responses wane over time even in as short a period as 6–7 weeks after symptoms onset (Ibarrondo et al., 2020; Long et al., 2020; Seow et al., 2020). Importantly our data shows the presence of SARS-CoV-2 memory T cells in convalescent donors with mild symptoms, which has enormous implications for protection against further SARS-CoV-2 infections and in decreasing the severity of COVID-19. Further studies with larger cohorts are needed to determine the SARS-CoV-2 memory duration and thereby elucidate the long-term protection to SARS-CoV-2, as has been previously demonstrated for another coronavirus (Ng et al., 2016).

For proper T-cell recognition, both the donor and recipient need to share HLA alleles. Given the vast number of convalescent donors, finding the proper haploidentical donor based on HLA typing would not be difficult. Based on previously published data (Leung et al., 2020), this donor can cover around 93.6% of Spain’s population. In accordance with the HLA donor-recipient match, we estimate that four donors will cover almost the whole of Spain’s population (Leen et al., 2013; Leung et al., 2020). These cells are expected to remain in the patients until the host immune system is recovered. Previous experience with HSCT has shown that these cells can be detected in the host for weeks (Triplett et al., 2015). Besides data from the phase I clinical trial (unpublished) shows that we can detect donor chimerism for 3 weeks.

The procedure for obtaining the cells is easy to implement for small-scale manufacture, is quick and cost-effective, and involves minimal manipulation. AlsoCD45RA^–^ memory T cell-based therapy is manufactured under the quality standards that apply to blood banks that perform HSCT daily with complex manipulations that are not considered advanced therapy medicinal product and can therefore be obtained without GMP condition requirements. The manufacturing of CD45RA^–^ memory T cells is carried out in closed automated systems similar to clean rooms that guarantee an aseptic process for the administration to the patient. These factors make it feasible to create a biobank or stock from the blood of convalescent donors, which would be immediately available “off the shelf” for subsequent outbreaks, increasing the therapeutic options in the current SARS-CoV-2 pandemic. A clinical trial is currently assessing the safety of these cells for patients with moderate to severe COVID-19 (NCT04578210).

These cells could provide patients with (1) a pool of SARS-CoV-2-specific T cells that will respond quickly to the infection, (2) a pool of cells for patients with severe disease presenting with lymphopenia, and (3) a pool of specific memory T cells for other pathogens from the donors encountered during their life, which are vital for eliminating other secondary infections that usually develop in patients hospitalized with COVID-19 (Kim et al., 2020; Zhu et al., 2020).

## Data Availability Statement

The raw data supporting the conclusions of this article will be made available by the authors, without undue reservation.

## Ethics Statement

The studies involving human participants were reviewed and approved by the Hospital Ramon y Cajal, Madrid, Spain. The patients/participants provided their written informed consent to participate in this study.

## Author Contributions

CF, BS, and AP-M designed the study. CF, BP-M, and CM-D performed the *in vitro* experiments. JV, AB, and FG-S performed the HLA typing and TCR spectratyping. RD and AM performed the non-mobilized apheresis and cryopreservation of the cells. CM-C performed the statistics. CF, BP-M, BS, and AP-M wrote the first draft of the manuscript. All authors revised the manuscript, participated in the interpretation of the data and the approval and submission of the manuscript.

## Conflict of Interest

CF, BS, and AP-M filed a patent on this topic. BS received fees from Celgene, Gilead, Sanofi and Novo-Nordisk unrelated to this work. The remaining authors declare that the research was conducted in the absence of any commercial or financial relationships that could be construed as a potential conflict of interest.

## Supplementary Material

Supplementary Figure 1 HLD-DR expression in the CD45RA^–^CD3^+^, CD45RA^–^CD3^+^CD4^+^, and CD45RA^–^CD3^+^CD8^+^ populations from the convalescent donor. Cells were cultured with 50 ng/mL of IL-15 o/n and for 72 h.

Supplementary Figure 2 CD69 expression in the CD45RA^–^CD3^+^, CD45RA^–^CD3^+^CD4^+^, and CD45RA^–^CD3^+^CD8^+^ populations from the convalescent donor. Cells were cultured with 50 ng/mL of IL-15 o/n and for 72 h.

Supplementary Figure 3 CD25 expression in the CD45RA^–^CD3^+^, CD45RA^–^CD3^+^CD4^+^, and CD45RA^–^CD3^+^CD8^+^ populations from the convalescent donor. Cells were cultured with 50 ng/mL of IL-15 o/n and for 72 h.

Supplementary Figure 4 NKG2A expression in the CD45RA^–^CD3^+^, CD45RA^–^CD3 + CD4^+^,and CD45RA^–^CD3^+^CD8^+^ populations from the convalescent donor. Cells were cultured with 50 ng/mL of IL-15 o/n and for 72 h.

Supplementary Figure 5 PD-1 expression in the CD45RA^–^CD3^+^, CD45RA^–^CD3^+^CD4^+^, and CD45RA^–^CD3^+^CD8^+^ populations from the convalescent donor. Cells were culture with 50 cultured/mL of IL-15 o/n and for 72 h.

Supplementary Figure 6 CCR7 expression in the CD45RA^–^CD3^+^, CD45RA^–^CD3^+^CD4^+^, and CD45RA^–^CD3^+^CD8^+^ populations from the convalescent donor. Cells were cultured with 50 ng/mL of IL-15 o/n and for 72 h.

Supplementary Figure 7 CD103 expression in the CD45RA^–^CD3^+^, CD45RA^–^CD3^+^CD4^+^, and CD45RA^–^CD3^+^CD8^+^ populations from the convalescent donor. Cells were cultured with 50 ng/mL of IL-15 o/n and for 72 h.
