# Supplementary figures and images for: SARS-CoV-2-Specific Memory T Lymphocytes From COVID-19 Convalescent Donors: Identification, Biobanking, and Large-Scale Production for Adoptive Cell Therapy
Source: Front Cell Dev Biol. 2021 Feb 25;9:620730. doi: 10.3389/fcell.2021.620730 (PMC7947351; doi:10.3389/fcell.2021.620730)

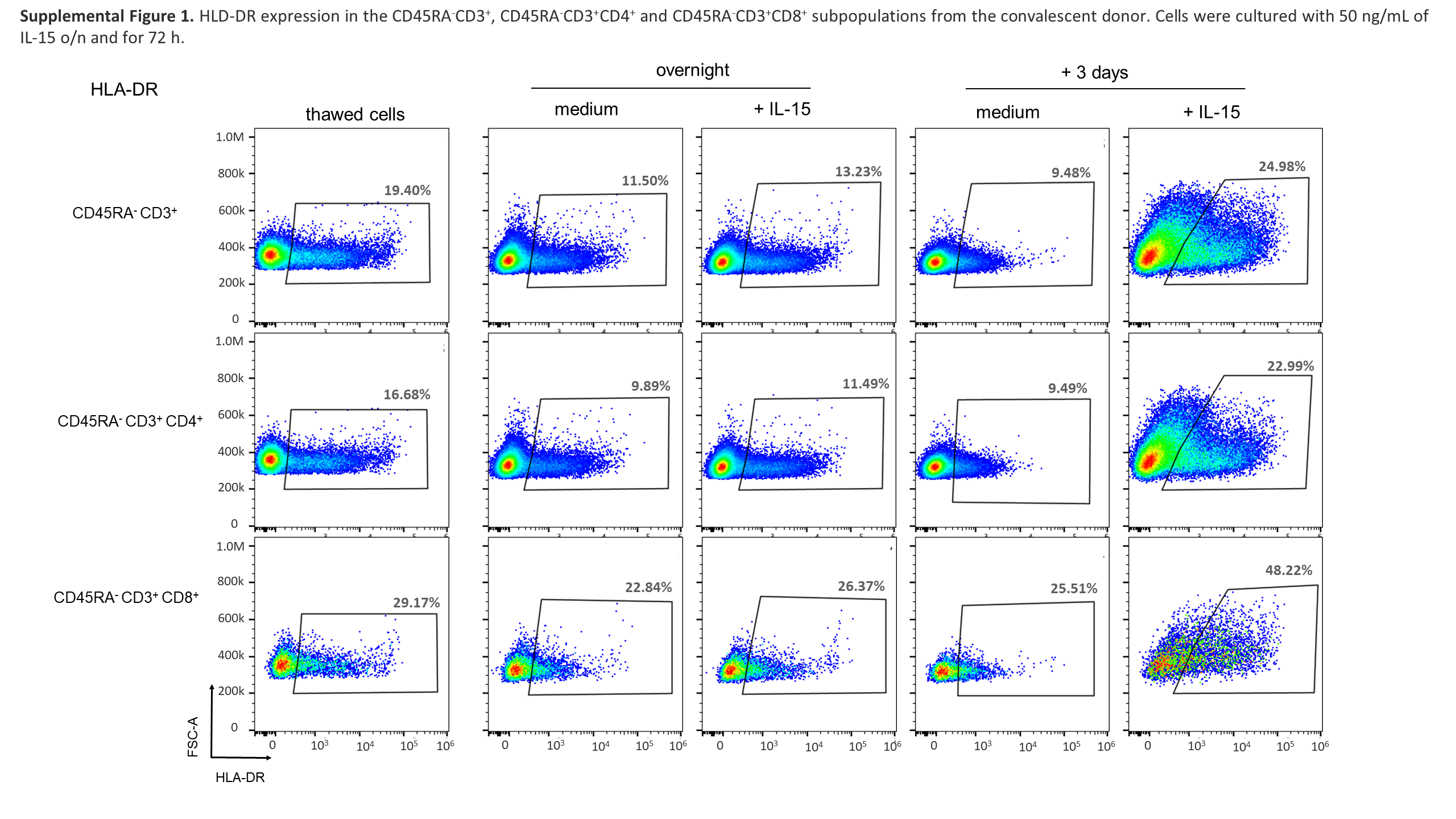

Supplement: Supplementary Figure 1 — HLD-DR expression in the CD45RA–CD3+, CD45RA–CD3+CD4+, and CD45RA–CD3+CD8+ populations from the convalescent donor. Cells were cultured with 50 ng/mL of IL-15 o/n and for 72 h. [file Image_1.TIF]

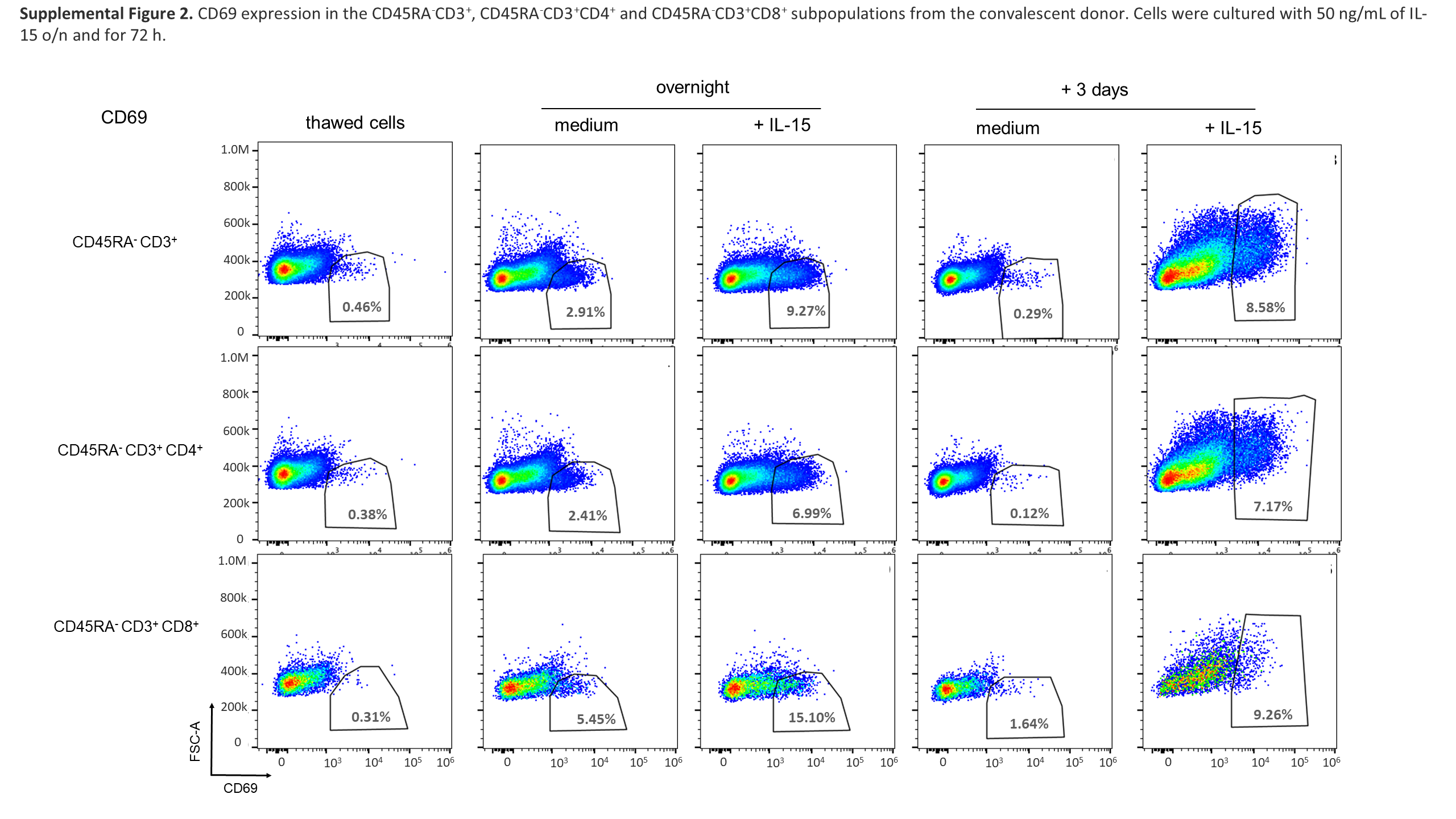

Supplement: Supplementary Figure 2 — CD69 expression in the CD45RA–CD3+, CD45RA–CD3+CD4+, and CD45RA–CD3+CD8+ populations from the convalescent donor. Cells were cultured with 50 ng/mL of IL-15 o/n and for 72 h. [file Image_2.TIF]

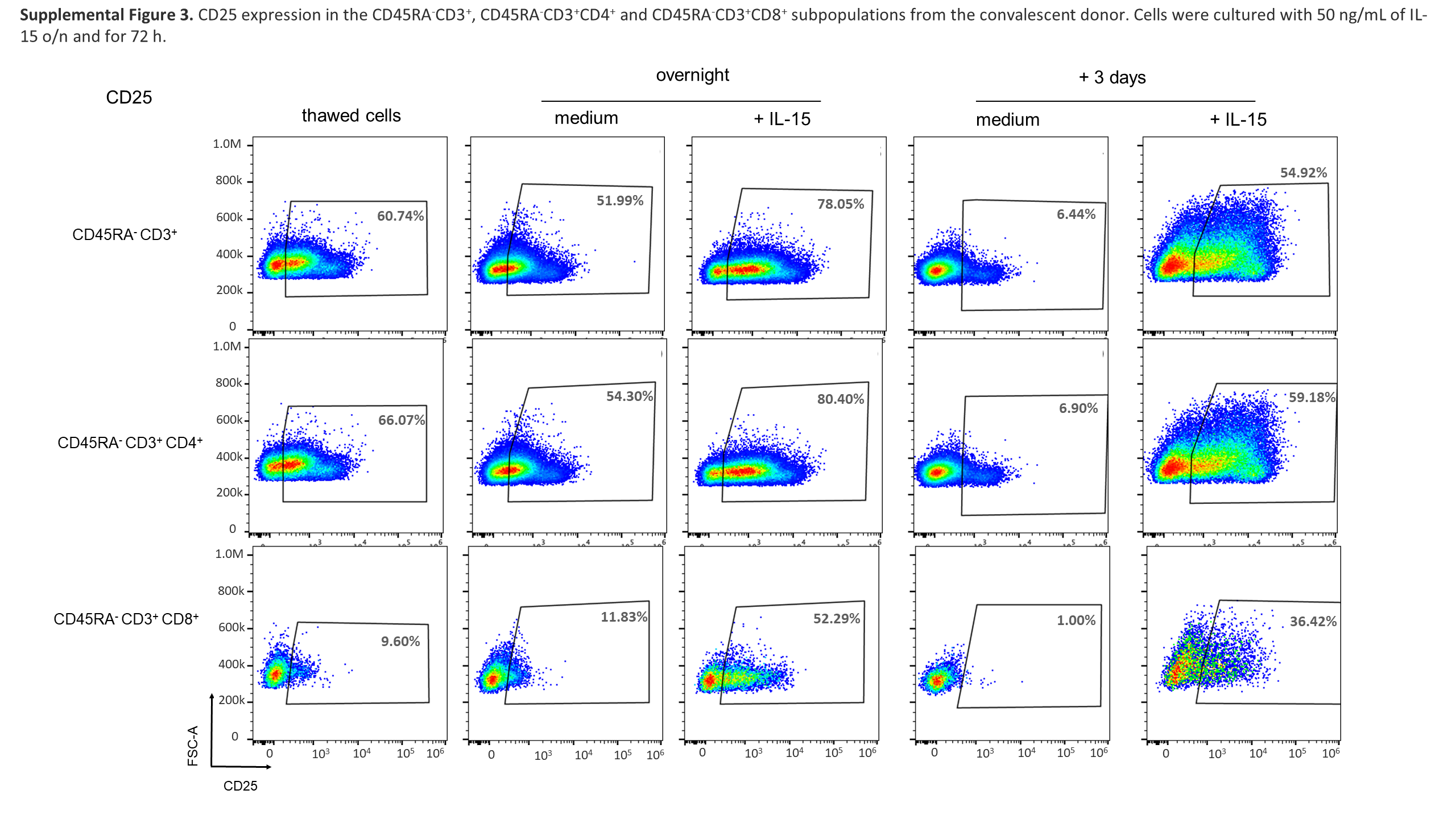

Supplement: Supplementary Figure 3 — CD25 expression in the CD45RA–CD3+, CD45RA–CD3+CD4+, and CD45RA–CD3+CD8+ populations from the convalescent donor. Cells were cultured with 50 ng/mL of IL-15 o/n and for 72 h. [file Image_3.TIF]

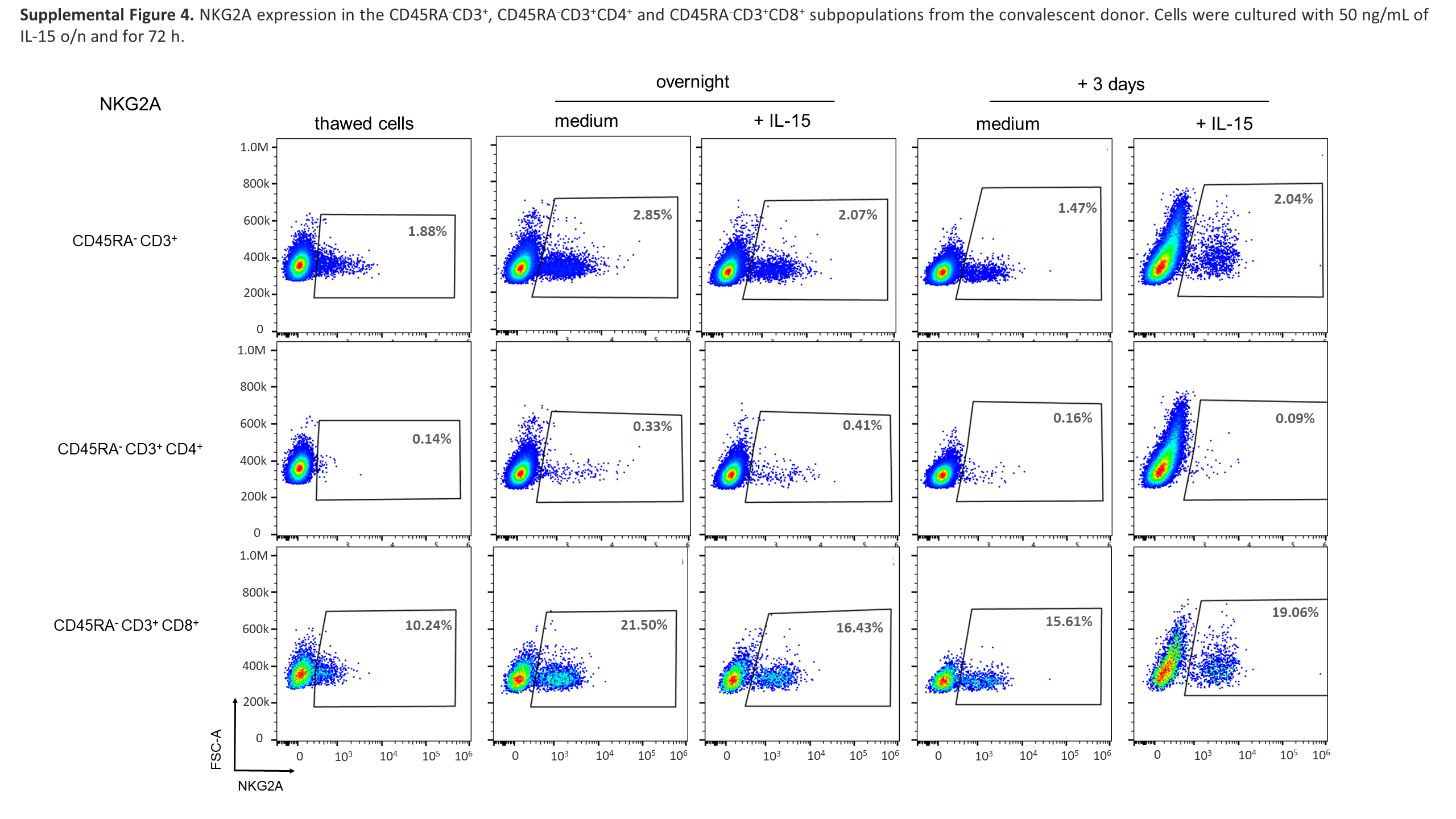

Supplement: Supplementary Figure 4 — NKG2A expression in the CD45RA–CD3+, CD45RA–CD3 + CD4+,and CD45RA–CD3+CD8+ populations from the convalescent donor. Cells were cultured with 50 ng/mL of IL-15 o/n and for 72 h. [file Image_4.TIF]

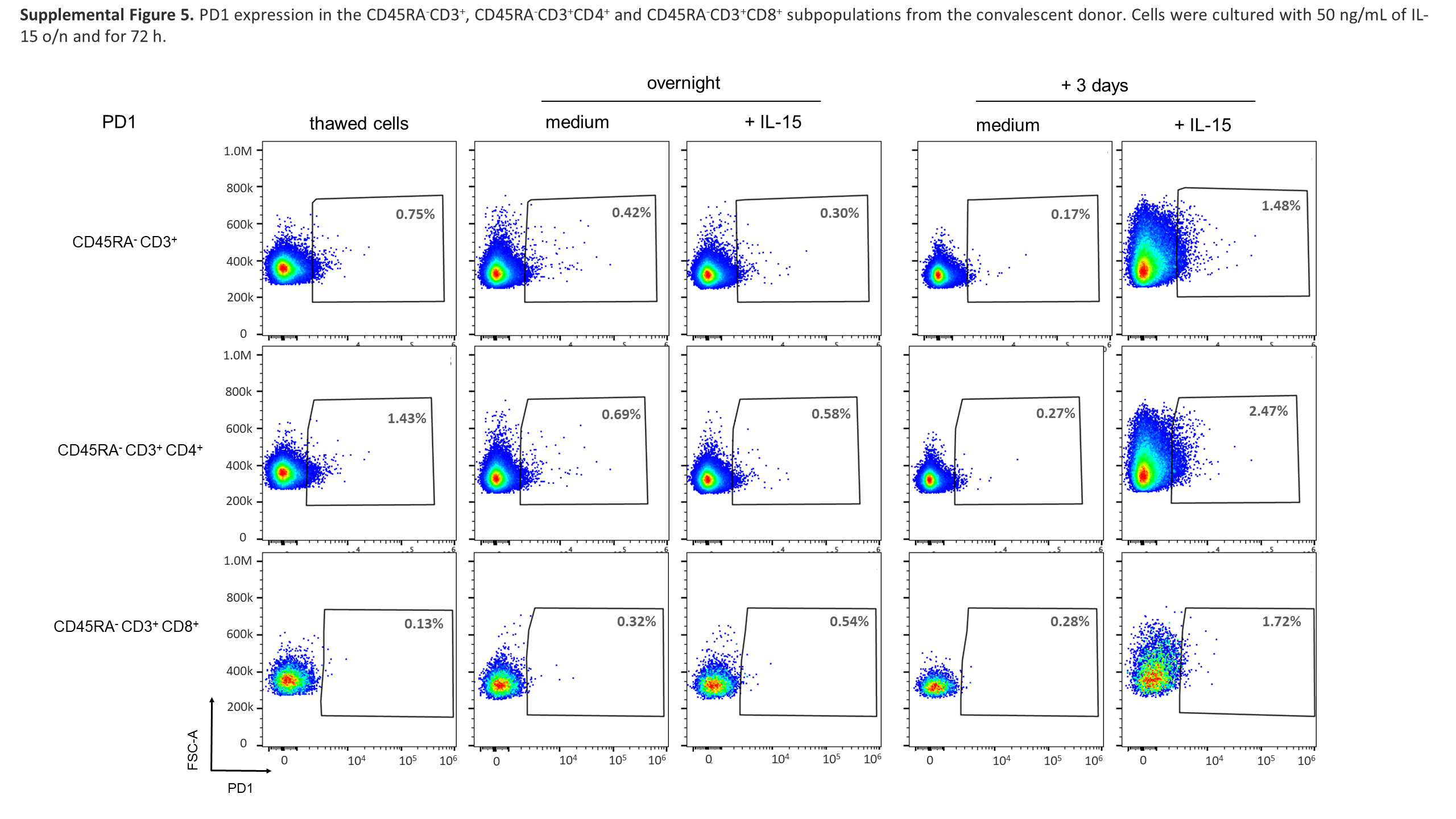

Supplement: Supplementary Figure 5 — PD-1 expression in the CD45RA–CD3+, CD45RA–CD3+CD4+, and CD45RA–CD3+CD8+ populations from the convalescent donor. Cells were culture with 50 cultured/mL of IL-15 o/n and for 72 h. [file Image_5.TIF]

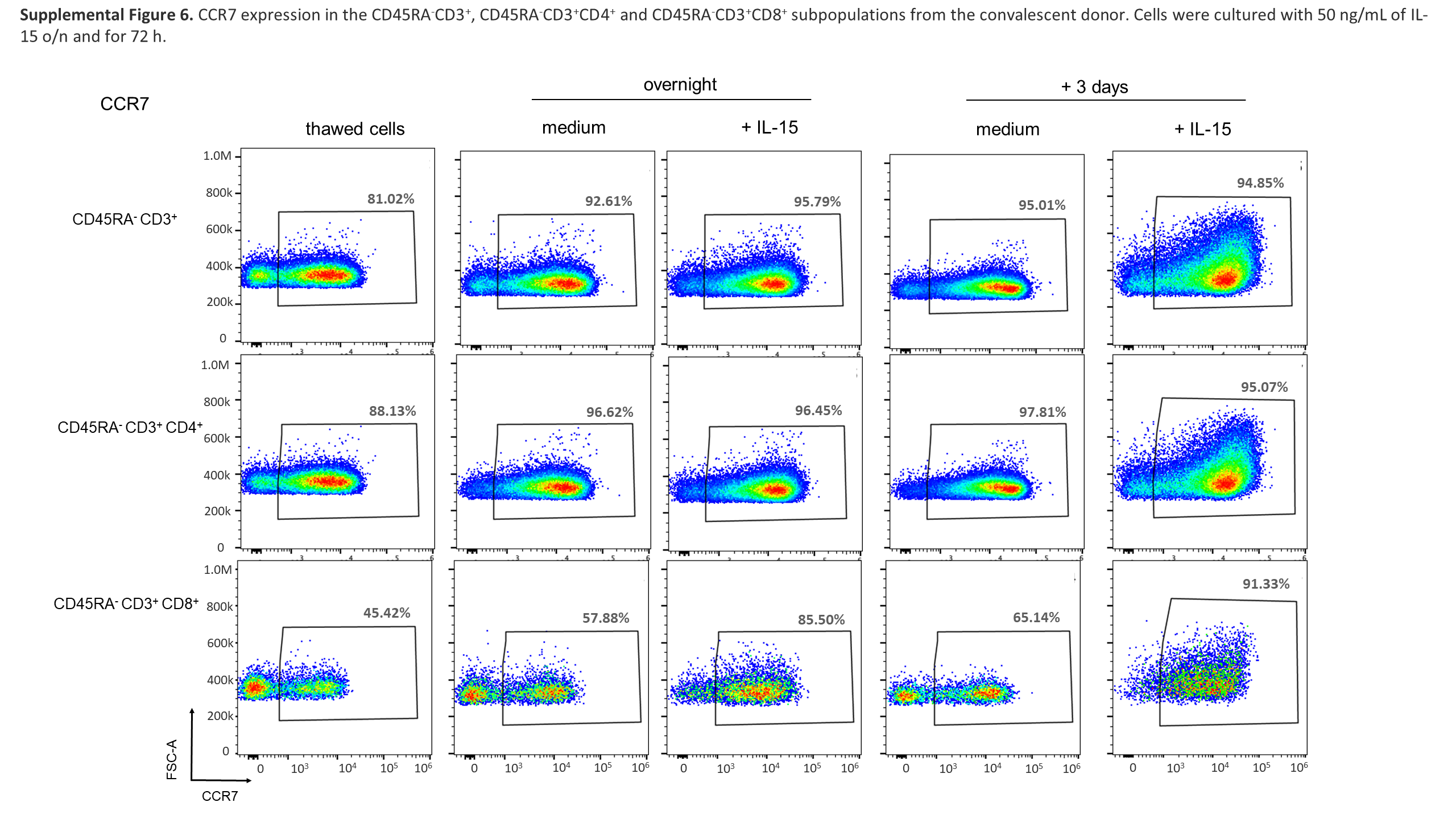

Supplement: Supplementary Figure 6 — CCR7 expression in the CD45RA–CD3+, CD45RA–CD3+CD4+, and CD45RA–CD3+CD8+ populations from the convalescent donor. Cells were cultured with 50 ng/mL of IL-15 o/n and for 72 h. [file Image_6.TIF]

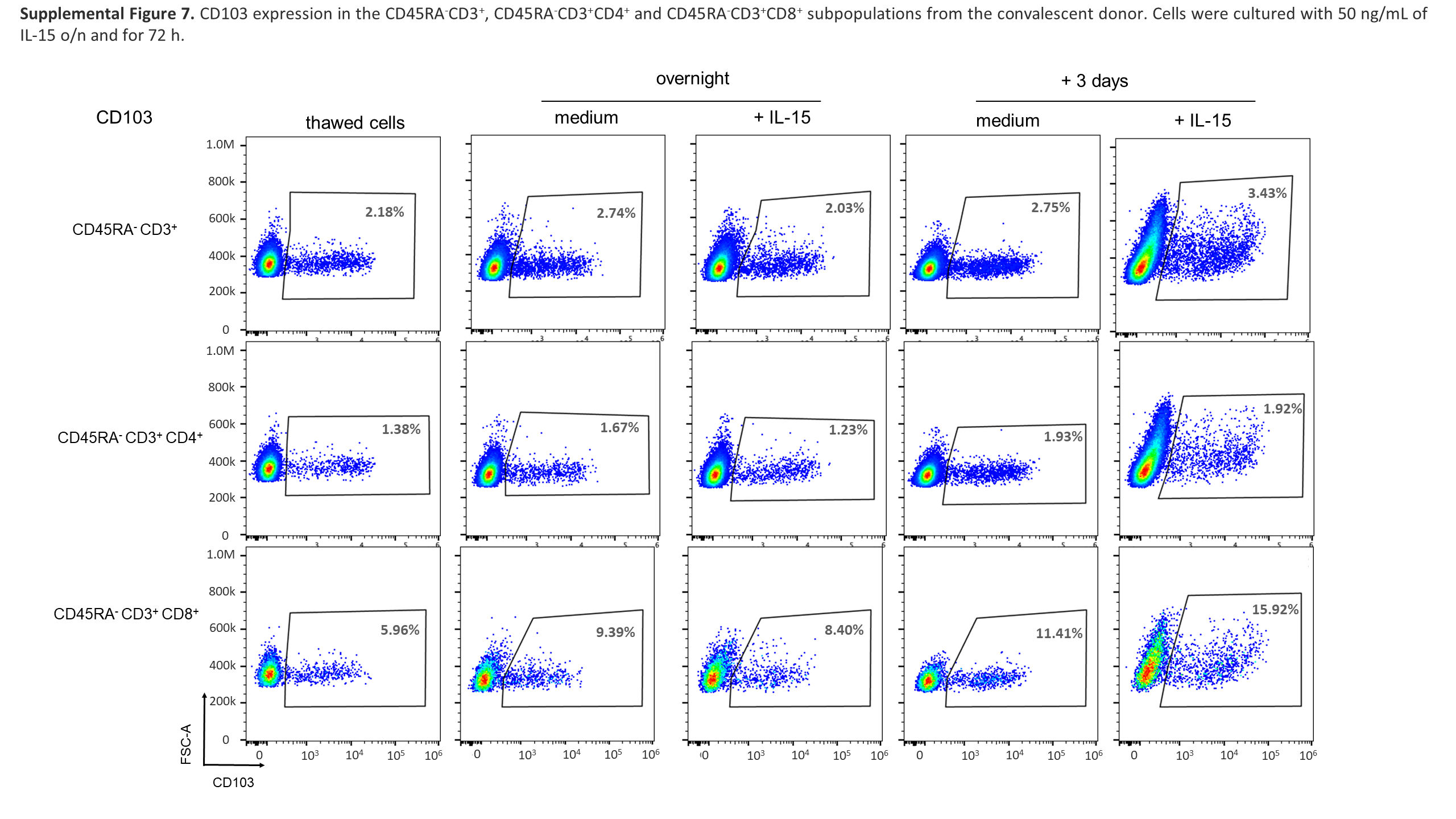

Supplement: Supplementary Figure 7 — CD103 expression in the CD45RA–CD3+, CD45RA–CD3+CD4+, and CD45RA–CD3+CD8+ populations from the convalescent donor. Cells were cultured with 50 ng/mL of IL-15 o/n and for 72 h. [file Image_7.TIF]
